# Linear Momenta Transferred to the Dental Implant-Bone and Natural Tooth—PDL-Bone Constructs Under Impact Loading: A Comparative *in-vitro* and *in-silico* Study

**DOI:** 10.3389/fbioe.2020.00544

**Published:** 2020-06-12

**Authors:** Ayda Karimi Dastgerdi, Gholamreza Rouhi, Mohammad Mehdi Dehghan, Saeed Farzad-Mohajeri, Hamid Reza Barikani

**Affiliations:** ^1^Faculty of Biomedical Engineering, Amirkabir University of Technology, Tehran, Iran; ^2^Department of Surgery and Radiology, Faculty of Veterinary Medicine, University of Tehran, Tehran, Iran; ^3^Institute of Biomedical Research, University of Tehran, Tehran, Iran; ^4^Dental Implant Research Center, Dentistry Research Institute, Tehran University of Medical Sciences, Tehran, Iran

**Keywords:** dental trauma, dental implant, periodontal ligament, impact loading, finite element analysis, linear momentum

## Abstract

During dental trauma, periodontal ligament (PDL) contributes to the stability of the tooth-PDL-bone structure. When a dental implant is inserted into the bone, the dental implant-bone construct will be more prone to mechanical damage, caused by impact loading, than the tooth-PDL-bone construct. In spite of the prevalence of such traumas, the behavioral differences between these two constructs have not been well-understood yet. The main goal of this study was to compare the momentum transferred to the tooth-PDL-bone and dental implant-bone constructs under impact loading. First, mechanical impact tests were performed on six canine mandibles of intact (*N* = 3) and implanted (*N* = 3) specimens using a custom-made drop tower apparatus, from release heights of 1, 2, and 3 cm. Next, computed tomography-based finite element models were developed for both constructs, and the transferred momenta were calculated. The experimental results indicated that, for the release heights of 1, 2, and 3 cm, the linear momenta transferred to the dental implant-bone construct were 33.1, 31.0, and 27.5% greater than those of the tooth-PDL-bone construct, respectively. Moreover, results of finite element simulations were in agreement with those of the experimental tests (error <7.5%). This work tried to elucidate the effects of impact loading on the dental implant-bone and tooth-PDL-bone constructs using both *in-vitro* tests and validated *in-silico* simulations. The findings can be employed to modify design of the current generation of dental implants, based on the lessons one can take from the biomechanical behavior of a natural tooth structure.

## Introduction

Dental trauma is one of the most prevalent occurrences in the field of dentistry, which could happen through falls, sports, motor vehicle accidents, and assaults that may cause damage to the dental structure (Bastone et al., 2000; Glendor, 2008; Andreasen et al., 2018). Severity of the damage to the dental structure induced by trauma depends on the location, intensity, direction of impact load, and also mechanical properties of the tooth, and its surrounding tissues, including the periodontal ligament (PDL) (da Silva et al., 2013). Dental trauma can occur directly or indirectly; the former takes place by a direct blow to the tooth, while the latter happens when the mandible is forcefully closed against the maxilla by a blow to the chin (Andreasen et al., 2018). Dental implants have been widely utilized as a replacement for missing teeth, and can be a concern in the case of trauma. This is due to the fact that a dental implant-bone (DI-B) construct differs significantly from a tooth-PDL-bone (T-PDL-B) construct, from both biological and engineering points of view.

The functionality of a dental implant has been reported to be correlated with the mechanical stresses transferred to the osseous tissue adjacent to the dental implant (Hansson, 1999; Chou et al., 2008). Loading type, implant geometry, and DI-B interaction have been shown to determine the mechanical load transferred from the dental implant to the surrounding bone (Hansson, 1999; Geng et al., 2001). Osseointegration, i.e., structural and functional connection between bone and the surface of an implant, is a key factor by which the stability of a dental implant can be measured (Geng et al., 2001). From a biomechanical point of view, an implant is considered osseointegrated if, under functional loading, there is no relative motion between the implant and the surrounding bone. Primary stability, i.e., initial mechanical engagement between the surface of the implant and bone of the osteotomy, is one of the perquisites for achieving osseointegration. The primary stability is gradually replaced with the secondary stability, provided by new bone formation, occurring during the healing process, which anchors the implant to the neighboring bone (Raghavendra et al., 2005).

Several experimental and numerical studies have investigated the biomechanical response of T-PDL-B and DI-B under various physiological and orthodontic loading conditions, including both static/quasi-static and dynamic loading (Clement et al., 2004; Cattaneo et al., 2009; Field et al., 2009; Chen et al., 2014, 2015). However, a few studies have investigated the effects of impact or high loading rate on T-PDL-B due to its lower prevalence during normal physiological condition, as well as inherent difficulties associated with experimental investigation of trauma (Fabra-Campos et al., 1991; Casas et al., 2007). Numerical methods, such as finite element analysis (FEA), have been employed as a felicitous approach to investigate traumatic dental damage, and to assess tooth behavior under traumatic impact loading (Huang et al., 2005, 2006; Miura and Maeda, 2008; da Silva et al., 2013). However, in spite of the prevalence of dental trauma to both T-PDL-B and DI-B, enough attention has not been paid to the differences in alveolar bone behavior surrounding natural teeth and dental implants, when impact loading occurs.

During static loading, the PDL which connects the tooth to the mandible/maxilla, acts as a supportive layer due to its soft structure and shock absorbing properties. This reduces the force transferred from tooth to the adjacent bone (Menicucci et al., 2002; Wang et al., 2015). It has been shown that the energy storage of PDL is about 161.5 J/mm^3^ at the time of loading and one-tenth of the stored energy is dissipated at the time of unloading during mastication (Pei et al., 2018). However, when a dental implant is used, there is no PDL between the implant and neighboring bone, and the implant is directly connected to the bone, and thus this causes alteration in the mechanical stimuli, e.g., stress or strain, distribution in the surrounding bone (Wang et al., 2015; Robinson et al., 2019). Experimental evidence has shown that PDL sustains large deformation and exhibits non-linear and time-dependent behavior, and thus hyper-elastic, visco-elastic, and visco-hyperelastic constitutive models have been used to describe its mechanical behavior (Zhurov et al., 2007; Huang et al., 2017). In addition to the importance of the PDL in static loading condition, its role is more crucial in the case of dynamic loading, such as trauma and impact loading, due to its time-dependent and non-linear behavior (Huang et al., 2005, 2006; da Silva et al., 2013). Nonetheless, due to short time duration of trauma, viscoelastic behavior of PDL can be reasonably neglected (Huang et al., 2005; da Silva et al., 2013). In impact loading, the PDL has been suggested to act as a shock absorber, which can reduce the energy transferred through impact, and thus mitigate the maximum stress exerted on the surrounding bone. In the DI-B construct, even in the ideal case, when the implant gets fully connected to the surrounding bone through complete osseointegration, which rarely happens (Geng et al., 2001), the biomechanical behavior of the DI-B construct will be different from that of the T-PDL-B construct. The existing differences between dampening capacity of natural dental systems and dental implants have fostered the researchers to develop new materials and designs for dental restoration, which can have the ability to mimic the natural behavior of dental systems to replace, or restore their function (Maminskas et al., 2016; Preis et al., 2017; Madeira et al., 2019).

The main goal of this study was to identify the main differences in linear momenta transferred to the T-PDL-B and dental DI-B constructs under impact loading. To accomplish this objective, first *in-vitro* impact tests were performed on six T-PDL-B and DI-B samples extracted from canine mandibles. A computed tomography (CT)-based FE model was then developed to assess the linear momenta transferred to DI-B and T-PDL-B complexes under the same impact loading condition. The boundary and loading conditions of the FE model were as close as possible to those of the experimental tests. Finally, through comparing the FEA results with the experimental data, a validated FE model was developed and employed to find the stress distribution within the constructs, as well as for further and future investigations on the DI-B and T-PDL-B constructs.

## Materials and Methods

**Figure 1:**
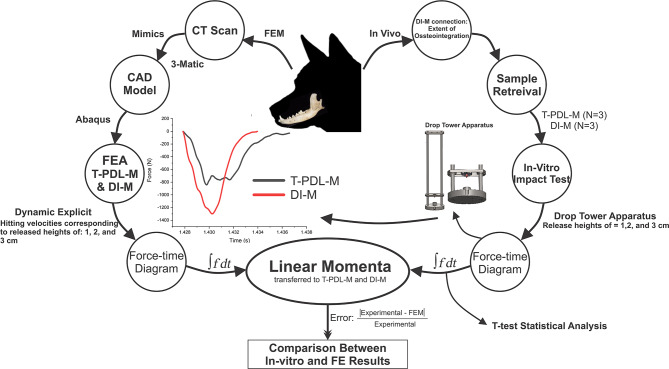
Schematic block diagram of the experimental set-up and FEA procedure.

This study was divided into three main phases: *in-vivo* experiments, *in-vitro* experiments, and finite element analyses. Tooth and dental implant samples for the *in-vitro* experiments, and data needed for the FE models, were obtained from the first step, i.e., *in-vivo* phase. Using a custom-made drop tower apparatus, impact loading was exerted on the extracted samples, the FE models were then developed based on the CT images of one of the samples, and boundary conditions were adapted from the *in-vitro* experiments (Figure 1).

### *In-vivo* Experiments

Three healthy mixed-breed dogs, aged 1–2 years, with an average weight of 22.7 kg were used in this study. They were housed in environmentally controlled rooms in the Faculty of Veterinary Medicine, University of Tehran, accredited as an animal care facility. Throughout the experiments, all dogs were fed commercial dry dog food and fresh water was provided. Imaging was performed by SIEMENS/Spirit CT device (resolution: 0.28 × 0.28*mm*^2^, slice thickness: 1 mm). The subject was anesthetized by intravenous injection of Ketamine and Diazepam, placed in a ventral position on the table, and then images were taken from the nose to the neck. A specific fixture was utilized to fix the oral cavity in an open position during the imaging.

**Figure 2:**
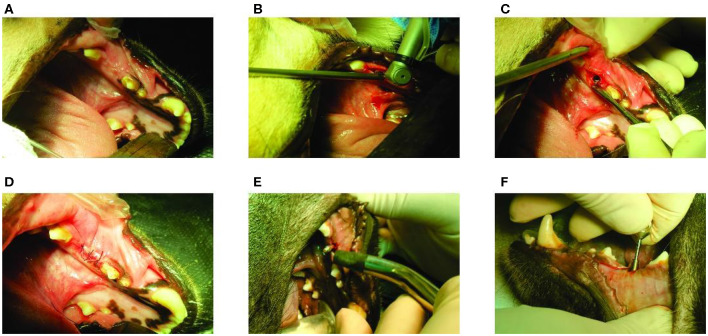
*In-vivo* experiments: **(A)** The extraction site after 8 weeks of healing; **(B)** Implant placement procedure; **(C)** Positioning of implant in the premolar area; **(D)** Suturing the implanted site using a 3–0 Polyglycolic Acid (PGA) suture; **(E)** Measuring implant stability through resonance frequency analysis (RFA) (Osstell® ISQ; Sweden); and **(F)** Mounting straight abutment (Bionic®, dental implants, Nickashtasia Inc.; Ti6Al4V, 4.5 mm of diameter, 2 mm of gingival height, & 7 mm of height) on the implant.

The surgery and peri-operative work dealing with the canines were performed in accordance with protocols approved by the Committee on Animal Care at Faculty of Veterinary Medicine at the University of Tehran. The surgeries for tooth extraction were performed under general anesthesia. Prior to extraction, the mouth rinsed with 0.05 chlorhexidine gluconate to decrease oral bacterial flora. A mucogingival flap with vertical releasing incisions at the mesial-buccal line angle of adjacent tooth was created. A tapered bur on a high-speed electric handpiece was used to section the third mandibular premolar, and each root was extracted separately. Luxators and elevators were used to cut and sever periodontal attachments. Once significant mobility has been achieved, extraction forceps were applied and each root was delivered from its alveolus. The empty alveolus was flushed with sterile saline solution, and the flap was repositioned and sutured without excessive tension, using a 3-0 Polyglycolic Acid (PGA) suture. The extraction sites were then allowed to heal for 8 weeks. Meloxicam and tramadol were administered pre-operatively as analgesia agents. Soft diet was provided for the first few days after the tooth extraction, and the extraction sites were checked daily until the wounds were completely healed. There were no signs of infection, and all extraction sites healed without any complication (Figure 2A).

After 8 weeks of tooth extraction, i.e., after complete healing, the implants were placed by an implantologist. The surgeries were performed under general anesthesia. A dental implant (Bionic®, dental implants, Nickashtasia Inc.; Ti6Al4V, 3.75 mm of diameter & 10 mm of length) was placed on the left side of the mandible of each animal and the right sides remained intact. Incision on the implant sites were performed using a #12 blade to gain access to the alveolar bone. Implant osteotomy sites were prepared at drilling speed of 1,100 rpm, using twist drills of 2.2, 2.8, and 3.2 diameters as pilot drill and the main drills, respectively (Figure 2B). The implants were placed with an insertion torque of 15 N.cm (Figure 2C). After implant placement, the oral mucosa over the implants was sutured using 3–0 PGA to cover the implants, and the implant sites were allowed to heal for 8 weeks before retrieval (Figure 2D).

Three main steps were taken to prepare the samples for *in-vitro* tests: measuring implant stability; placing abutment; and retrieving specimens. After the designated healing period, i.e., 8 weeks, osseointegration was evaluated using resonance frequency analysis (RFA) (Osstell® ISQ; Sweden), and all the measurements were followed according to the manufacturer's instructions (Figure 2E). The implant stability quotient (ISQ) values were measured three times on each implant, and their average value were found to be 64, 66, and 72, which are all within the acceptable clinical range (Cehreli, 2012). After ensuring the implants stability, the animals were euthanized by an intravenous injection of sodium thiopental and straight abutments (Bionic®, dental implants, Nickashtasia Inc.; Ti6Al4V, 4.5 mm of diameter, 2 mm of gingival height, & 7 mm of height) were manually mounted on the screws by a hand wrench (Figure 2F). Since the impact loading was applied by a flat and smooth disc on the top surface of teeth and dental implants, it was necessary to reduce the heights of adjacent teeth on both sides of the mandible. To do this, the adjacent teeth to the third premolars, and to the dental implants were carefully shortened by means of a turbine. Finally, bilateral mandibles including implanted and intact parts were harvested by the surgeon, and the samples were stored in 10% formalin for 24 h before *in-vitro* tests.

### *In-vitro* Experiments

**Figure 3:**
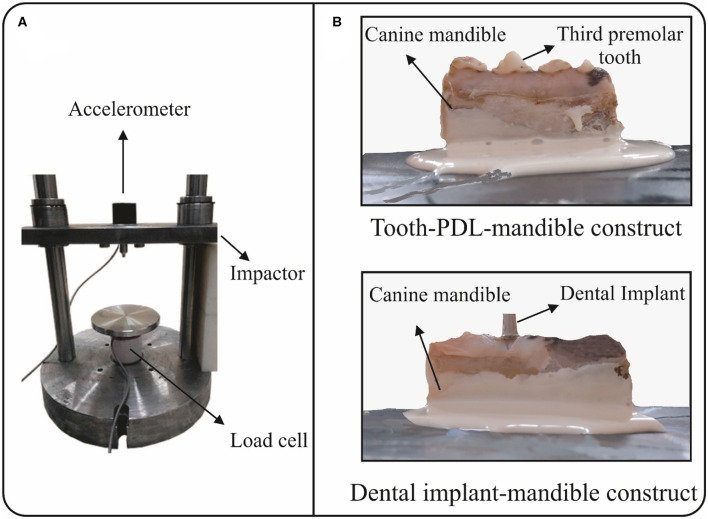
*In-vitro* experimental set-up: **(A)** A custom-made drop tower apparatus used to mimic impact loading, which contains three main parts: an accelerometer, a load cell, and a flat and smooth impactor; and **(B)** Fixing the inferior surface of the specimens, i.e., tooth-PDL-mandible and dental implant-mandible constructs, by stone adhesive and Mitreapel BK-1002 adhesive on steel plates.

A custom-made drop tower apparatus was used to mimic impact loading in this study. The apparatus is composed of three main parts: a flat and smooth impactor with a mass of 2.5 kg; an accelerometer (ACX-500-KU), which is mounted on the impactor; and a load cell on which the specimen is placed (Figure 3A). Different impact loadings were induced by altering the height of releasing the impactor over the specimen. The accelerometer, which records acceleration of the impactor, can measure accelerations up to 500 g, i.e., about 5,000 m/s^2^, and has a bandwidth of 17 kHz. The load cell, which records the impact load, can measure up to 20 kN. Acceleration-time and load-time data, which were recorded during impact by the accelerometer and load cell, respectively, were transmitted to a computer via a 100 kHz data transmitter. Two different groups of intact and implanted constructs, i.e., tooth-PDL-mandible (T-PDL-M) and dental implant-mandible (DI-M), were used in *in-vitro* experiments, and three specimens were included in each group. In order to place the specimens into the impact apparatus, six steel sheets, with the area of 15 × 15 *cm*^2^, were prepared. The inferior surface of all specimens were fixed by stone adhesive and Mitreapel BK-1002 adhesive (Figure 3B), which were fully dried before the tests. The tests were performed by dropping the impactor from the heights of 1, 2, and 3 cm for each specimen, respectively.

### Finite Element Modeling

**Figure 4:**
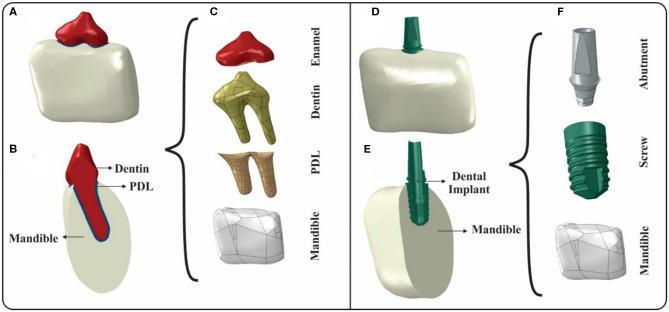
Generation of intact and implanted FE models: **(A)** Assembly of the tooth-PDL-mandible (T-PDL-M) construct in Abaqus software; **(B)** Cross-sectional view of T-PDL-M construct; **(C)** Constituents of T-PDL-M construct used to assemble the intact model; **(D)** Assembly of the dental implant-mandible (DI-M) construct in Abaqus software; **(E)** Cross-sectional view of DI-M construct; and **(F)** Constituents of DI-M construct used to assemble the implanted model.

Two FE models, based on the CT images taken from the specimens, one including T-PDL-M, the other one consisting DI-M, were made to study the effects of impact loading on them (Figure 4). The raw DICOM data was imported to Mimics (V 20.0, Materialize) to generate the 3D models. The 3D models of the third premolar tooth and a segment of the mandible were built separately for the intact construct. Since the surfaces of the constructed parts were rough, additional process was performed to smoothen them via 3-matic (V.12.0, Materialize). The tooth was considered to consist dentin and enamel, which was modeled as a skin on top of the tooth with a uniform thickness of 0.25 mm (Bath-Balogh et al., 1997). The PDL was generated around the root of the tooth with an average thickness of 0.2 mm (Figure 4C). For the implanted model, a computer aided design (CAD) of the different components of dental implant, i.e., screw and abutment were developed. Therefore, the implanted model included a segment of mandible, screw, and abutment (Figure 4F). Mesh generation was performed using 10-node tetrahedral elements, and the convergence tests for the intact and implanted models resulted in 469,212 and 608,993 elements, respectively.

The mechanical properties of the tooth, implant, and cortical bone used in FEA are listed in Table 1 (Huang et al., 2005; Ammar et al., 2011; Wang et al., 2015), and they were all considered to be homogenous, isotropic and linear elastic. The PDL is known to exhibit non-linear elastic behavior and published values for its Young's modulus show a large range from 0.01 to 100 MPa (Groning et al., 2011; Fill et al., 2012). Thus, in this study, in order to take into account the non-linear behavior of PDL under high loading rate, it was modeled as an isotropic, hyper-elastic material, 1^st^ order Ogden hyper-elastic model (Nikolaus et al., 2017) (Table 1).

**Table 1:** Mechanical properties of various constituents of tooth-PDL-mandible and dental implant-mandible constructs used in FE analysis (Huang et al., 2005; Ammar et al., 2011; Wang et al., 2015).

**Material**	**Young modulus (GPa)**	**Poisson ratio**	**Density ($\frac{\text{g}\text{r}}{{\text{c}\text{m}}^{3}}$)**
Enamel	77.9	0.33	3.00
Dentin	18.6	0.31	1.20
Cortical Bone	14.7	0.3	1.74
Titanium	114	0.34	4.51
Mechanical properties of periodontal ligament used in FE analysis (Nikolaus et al., 2017)			
1st order Ogden hyper-elastic model coefficients used for PDL			
	*D*_1_	**α_1_**	**μ_1_**
	0.1	6.4	0.4

The boundary conditions for all FE models were as close as possible to those of the experimental tests, and inferior surface of the mandible was restricted in all directions. The interfaces of the cortical bone-PDL, tooth-PDL, and abutment-screw were assumed to be perfectly bonded to avoid relative motion between them. Most FE models consider fully bonded interfaces between bone and implant to be mimicking the state of a complete osseointegration, meaning that the bone is perfectly bonded to the implant; however, this rarely happens in clinical situations (Brunski, 1992; Williams, 2005; Huang et al., 2008). To construct a more precise FE model for this study, a partial osseointegration with a friction coefficient of 0.45 was considered between implant and bone surfaces (Brunski, 1992; Williams, 2005). Moreover, in order to simulate the effects of the drop tower impact apparatus, an analytical rigid disc was designed with dimensions compatible to the impactor, and the impactor's weight (2.5 kg) was applied to the disc. In the *in-vitro* experiments, the release height of the impactor was used to calculate the hitting velocity of the impactor before collision, using the following equation: $v^{2}-{v_{0}}^{2}=2gh$ where “$v_{0}=0.\;g\approx 10\frac{m}{s^{2}}$”. The interface between the surface of the impactor and top of the implant and tooth was assumed to be frictionless, kinematic surface-to-surface.

### Experimental, Statistical, and FEM Analyses

Force-time graphs were extracted from the impact tests, which were equivalent to the transmitted force to the entire system, measured in the lower region of the specimen placed in the apparatus. Since the raw data was turbulent, the Fourier Series and a low pass filter, written in MATLAB (V2015b, MathWorks), were used which significantly reduced fluctuation in the data. The linear momentum, i.e., the enclosed area of force-time curve, was then calculated for all force-time graphs, and the data of linear momenta were reported as average ± standard deviation. The *T*-test analysis was then used to compare the transmitted linear momenta in DI-M and T-PDL-M constructs, and differences were considered to be statistically significant for *p* > 0.05.

In order to be able to compare FE analyses with those of the experimental results, the transmitted force-time graphs were extracted for both FE models, i.e., DI-M and T-PDL-M models, and their corresponding linear momenta were calculated. At release heights of 2 and 3 cm, the induced stress in the alveolar bone cavity surpasses the yield stress of cortical bone, based on the FEA results. Thus, the linear plastic properties were assigned to the alveolar bone of the T-PDL-M construct by using a compressive modulus of 133 MPa (Ammar et al., 2011). In the DI-M construct, based on the FEA results, the induced stresses in the abutment and alveolar bone cavity surpassed the yield stress of titanium and cortical bone, respectively. Therefore, the linear plastic properties were assigned to the abutment and alveolar bone of the DI-M construct, by using compressive moduli of 880 MPa and 133 MPa, respectively (Ammar et al., 2011). The dynamic explicit FE analysis was implemented in Abaqus (V 6.18, Simulia, Dassault Systems) for both T-PDL-M and DI-M constructs.

## Results

Based on *in-vitro* tests, the average of the linear momenta transferred to T-PDL-M construct was found to be significantly smaller than that of DI-M construct for the release heights of 1 and 3 cm (*P* < 0.05) (Table 2). The difference between the mean values of linear momenta transferred to the two constructs at release height of 1 cm was found to be 33.1%. However, for release height of 2 cm, the calculated *p* > 0.05, which satisfies the nullity of the difference between the momenta transferred to the two different constructs, hit by the impactor (Table 2). The mean values of linear momenta transferred to the two constructs had a difference of 31% for the release height of 2 cm. The mean values of linear momenta of the two constructs, i.e., T-PDL-M and DI-M, had a difference of 27.5% for the release height of 3 cm, and the *p*-value was found to be <0.05, indicating that there is a significant difference between the two constructs (Table 2).

**Table 2:** Results of *in-vitro* tests: Mean, standard deviation, difference of the transferred linear momenta (∫**F** **dt**), and *t*-test statistical analysis of mean values of linear momenta transferred to tooth-PDL-mandible and dental implant-mandible constructs due to impact loading from release heights of 1, 2, and 3 cm.

**Release height of impactor**	**1 cm**				**2 cm**				**3 cm**			
**Subject**	**T-PDL-M**	**DI-M**	**Difference**	***p*-value**	**T-PDL-M**	**DI-M**	**Difference**	***p*-value**	**T-PDL-M**	**DI-M**	**Difference**	***p*-value**
	**construct**	**construct**	**(%)**		**construct**	**construct**	**(%)**		**construct**	**construct**	**(%)**	
Linear Momentum (kg.m/s)	2.92 ± 0.34	4.37 ± 0.8	33.1	0.03	3.69 ± 0.7	5.36 ± 1.13	31	0.06	4.80 ± 0.7	6.62 ± 0.93	27.5	0.027

Linear momenta of all FE models were calculated using MATLAB and compared with the *in-vitro* test results. The data of FEA indicates that greater linear momenta were transmitted to the DI-M construct than that of the T-PDL-M, under the same impact loading, which is in agreement with the behavior observed in the *in-vitro* tests. Comparing the mean values of *in-vitro* and *in-silico* findings of the linear momenta transferred to the two constructs, for the same impact loading revealed that there is a small difference, i.e., 1.9–7.5%, between the *in-vitro* and *in-silico* approaches employed in this study, and thus the FE models can be verified (Table 3).

**Table 3:** Linear momenta transferred into tooth-PDL-mandible and dental implant-mandible constructs: Comparison between results of *in-vitro* tests and FE models.

**Release height of impactor**	**Tooth-PDL-mandible construct**			**Dental implant-mandible construct**		
	**Mean experimental**	**FEM linear**	**Error (%)**	**Mean experimental**	**FEM linear**	**Error (%)**
	**linear momentum (Kg.m/s)**	**momentum (Kg.m/s)**		**linear momentum (Kg.m/s)**	**momentum (Kg.m/s)**	
1 cm	2.92 ± 0.34	2.70	7.5	4.37 ± 0.8	4.26	2.5
2 cm	3.69 ± 0.7	3.76	1.9	5.36 ± 1.13	5.21	2.2
3 cm	4.80 ± 0.7	4.68	2.5	6.62 ± 0.93	6.23	5.9

**Figure 5:**
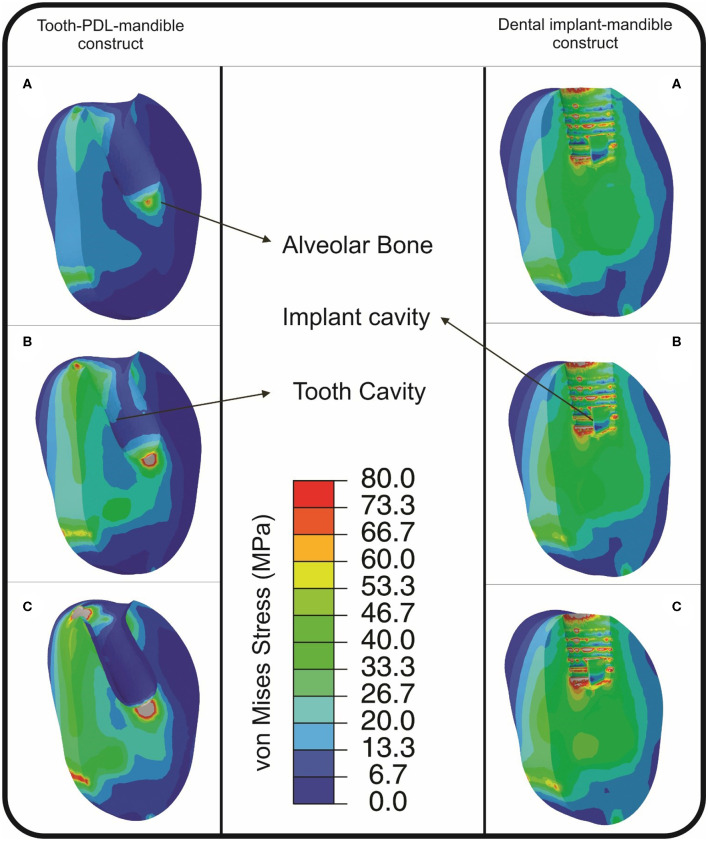
von Mises stress distribution (MPa), found from FE simulations, in alveolar bone in tooth-PDL-mandible (T-PDL-M) and dental implant-mandible (DI-M) constructs, resulted from impact loading simulations for the release heights of: **(A)** 1 cm; **(B)** 2 cm; and **(C)** 3 cm. The stress distribution indicates that the maximum induced stress in DI-M construct is more than that of T-PDL-M construct, in all cases. In T-PDL-M, stresses above 88 and 110 MPa in **(B,C)**, respectively, are shown with silver color, while in DI-M, stresses above 133, 198, and 283 MPa are shown with silver color, respectively, in **(A–C)**.

The von Mises stress distribution, which contains important information depending on its value and polarity for the alveolar bone of both constructs, was found using FE models. For the same impactor hitting speed, the induced von Mises stress in alveolar bone of the DI-M construct was found to be more than the T-PDL-M construct (Figure 5). At the release height of 1 cm, the induced stress in an implant cavity of DI-M construct surpassed the yield stress of cortical bone, i.e., 133 MPa (Ammar et al., 2011), and the whole system experienced plastic deformation (Figure 5A). Despite this, in the T-PDL-M construct, the system only experienced elastic deformation at the release height of 1 cm, and plastic deformation occurred only at the release heights of 2 and 3 cm (Figures 5B,C). As can be seen in Figure 5, for DI-M construct, alveolar bone experienced a high stress concentration inside the hole, while T-PDL-M resulted in much lower stress intensity. The stress contours also revealed that, as expected, the more speed the impactor has, the greater the induced stress will be in the alveolar bone in both constructs (Figure 5).

## Discussion

Despite the great importance and high occurrence of dental trauma, not enough attention has been paid to this crucial issue in the literature thus far. The finite element method (FEM) is now a well-accepted and a powerful tool in dental trauma research, due to many challenges associated with experimental works in this field (Huang et al., 2005, 2006; da Silva et al., 2013). This work was intended to discover the differences in the behavior of a dental implant-bone (DI-B) and tooth-PDL-bone (T-PDL-B) constructs, under impact loading. It was hypothesized that due to the presence of a soft, shock absorbing structure, i.e., PDL, between the tooth and neighboring bone, momentum transferred to the tooth-PDL-bone structure should be considerably less than that of dental implant- bone construct. Thus, the main scope of this work was to reveal the behavioral differences between a dental implant and a natural tooth under impact loading, and consequently to put an emphasis on the vital role of the PDL in the natural tooth, using *in-vivo, in-vitro*, and *in-silico* tools. The ultimate goal of this research was to take some lessons from the natural tooth construct in order to bio-mimetically modify currently designed, man-made teeth, i.e., dental implants.

In the *in-vitro* phase of this study, mechanical impact tests were performed on six extracted samples of canine mandibles at three release heights of 1, 2, and 3 cm. By recording transmitted force-time to the two constructs during impact, and calculating corresponding linear momenta, behavior of the two constructs under analysis, i.e., DI-M and T-PDL-M, were analyzed and compared with each other. In impact tests, significant differences were observed between the behavior of T-PDL-M construct and DI-M construct for the release heights of 1 and 3 cm (see Table 2). It is speculated that at the beginning of the tests, i.e., release height of 1 cm, the dental implant which was surrounded by bony tissue, due to osseointegration occurred after surgery (ISQ = 64, 66, and 72), had less micro-motion compared to the natural tooth, in which there was a soft tissue, i.e., PDL, between the tooth and mandible, and thus its behavior was totally different from the natural tooth under the same impact loading, and thus a much greater linear momentum was transferred to the alveolar bone of the former (see Table 2). However, for the second release height, i.e., h = 2 cm, since the first impact loading reduced the extent of stiff connection between the implant and neighboring bone, and thus caused a softer connection between the DI and neighboring bone, i.e., the case of partial osseointegration, its behavior was more similar to that of the natural tooth construct (see Table 2). For the third height, i.e., h = 3 cm, the reported p-value implied that the DI-M construct's behavior differed significantly from the T-PDL-M construct's behavior (Table 2). This can be due to a lack of connection or osseointegration, between the implant and neighboring bone, and thus most of the impact was transferred from the implant to the bone, in the case of DI-M, which is not the case in the T-PDL-M construct due to the presence of a shock absorber that causes dissipation of energy and reduces momentum transfer to the bone. The acceleration-time data, not shown in this paper, also indicated that less acceleration was produced in T-PDL-M construct, compared to that of the DI-M construct, which can be due to the presence of a shock absorber constituent in T-PDL-M construct, i.e., PDL, that causes dissipation of energy, and thus reduces the extent of mechanical damage to the bone.

In the *in-silico* simulation phase of this work, CT based finite element models of the mandible, including third premolar tooth and dental implant were developed (Figure 4). Impact loading was then simulated for the two constructs, i.e., DI-M and T-PDL-M, by applying an equivalent hitting speed of the impactor, calculated based on the release heights of the *in-vitro* tests. By eliciting transmitted force-time data, and calculating corresponding linear momenta, the FEM findings were compared with the experimental results for both constructs. The FEM data showed a good agreement with the experimental data collected in *in-vitro* tests, with an error of <7.5%, in all models (see Table 3). Differences between the boundary conditions of *in-vitro* tests and FEM, and also simplifications made in the *in-silico* models contribute to the discrepancies observed between the two approaches. For instance, the impact test is destructive, meaning that after the first impact loading, the specimen is weakened, micro-fractures most likely occur, and consequently in the next round of loading, the force values do not show a significant increase (see Table 2), whereas the FEM does not take into account the alterations occurred in various components of the system, and thus showed a noticeable increase in the force magnitude (see Table 3). Moreover, in the *in-vitro* tests, the implant of DI-M construct had a slight vertical displacement into the alveolar bone cavity due to impact loading, which eventually led to a transverse crack in the mandible. Whereas, in the FEM, the vertical displacement of the implant and damage of the mandible were not taken into account. In addition, in T-PDL-M construct, a crack was observed at enamel region for the release height of 3 cm, in the *in-vitro* tests, but in the FE simulation, that crack was not modeled, and the brittle behavior of enamel was not taken into account.

Considering the great importance of stress distribution within bone (Rouhi, 2011; Haase and Rouhi, 2013; Rouhi et al., 2015), von Mises stress distribution was found using FE simulation, for both T-PDL-M and DI-M constructs. In DI-M construct, the induced von Mises stress in the implant, also in alveolar bone cavity, indicated that the whole system underwent plastic deformation, even in the case of the minimum hitting speed, i.e., for the release height of 1 cm (see Figure 5A). Whereas, the induced von Mises stress in alveolar bone of the T-PDL-M construct showed that the mandible was subjected to plastic deformation just in the case of impact loading with the release heights of 2 and 3 cm (see Figures 5B,C). In all models, the maximum induced von Mises stresses in alveolar bone cavity of DI-M construct was greater than those of the T-PDL-M construct, which makes logical sense because of the presence of a dampening constituent in T-PDL-M construct, i.e., PDL (Figure 5), that dissipates energy given to the construct, and thus reduces deformation experienced by the alveolar bone. This finding is in agreement with some other studies, such as (Pietrzak et al., 2002; Wang et al., 2015; Pei et al., 2018), which have highlighted the role of PDL in the dissipation of biting forces during mastication. Results of the FE simulation of this work also revealed that the duration of impact loading was less in the DI-B construct compared to that of the T-PDL-B construct, with a higher peak of stress concentration in the former. Huang and co-workers showed that the time duration of impact loading increases for the tooth with a higher dampening property, and consequently the peak of stress-induced in the adjacent alveolar bone decreases (Huang et al., 2006). Thus, it can be concluded that an impacted tooth with a higher damping property, compared to a dental implant, can lower the concentrated stresses by dispersing the strain energy over a more extended time period.

Limitations of this work should be kept in mind when one is trying to interpret its outcomes. Firstly, the chosen release heights in impact apparatus, which were used to simulate the impact loading, was made due to the dearth of data about the methodology of impact loading for T-PDL-B and DI-B specimens. It was safe to start the impact loading from the release height of 1 cm, and then the release heights were increased incrementally until the failure of the samples occurred. Furthermore, since the scope of this study was to compare the behavior of the two constructs and consequently, both constructs should undergo the same experimental procedure. The average resultant peak forces in T-PDL-B construct, caused by the impact loading from the release heights of 1, 2, and 3 cm, were 792.8, 1109.5, 1612.0 N, respectively, which were in the same range as those reported in Huang et al. (2005), Huang et al. (2006), and da Silva et al. (2013).

Secondly, even though it is known that bone, tooth, and PDL are heterogeneous and anisotropic materials, similar to other studies existing in the literature related to modeling dental trauma, such as: Huang et al. (2005) and da Silva et al. (2013), they were assumed to be homogeneous, and isotropic materials in this study. Considering that disregarding anisotropy will result in an overestimation of the maximum stress induced in cortical bone, under physiological loading condition (O'Mahony et al., 2001), it is thought that more elaborative models are needed to obtain information on the significance of anisotropy on the analysis of the stress in T-PDL-B and DI-B constructs, under impact loading. Even though, based on the CT-images taken from the specimens, analogous size was chosen in order to eliminate the sensitivity of FE analysis to the variation of specimen's geometry in this study, non-etheless, it might be better to use specimen-specific FE models for each T-PDL-B construct. Another simplification made in this study was to disregard some of the constituents such as pulp and cementum, also to neglect variation in the PDL's thickness in the *in-silico* models. Mechanical damage caused by impact loading in the components of the constructs under analysis was also disregarded in our *in-silico* models.

Lastly, the low number of samples used was another limitation of this study. Moreover, deficiency of an impact apparatus capable of recording acceleration and force data forced us to use the impact apparatus with a fairly large dimensions, compared to the dimensions of the specimens, which could likely cause some errors. Furthermore, implant's crown was not used in this work, since there were some limitations in manufacturing of identical crowns to the canine teeth. In spite of the aforementioned limitations, it is thought that results of this study can improve our knowledge of the crucial role of the PDL in the emergence of behavioral differences between T-PDL-B and DI-B constructs, and can be deemed as a basis for future studies.

## Conclusions

This is the first study, to our best knowledge, which explores the effect of impact loading on tooth-PDL-bone and dental implant-bone constructs using both *in-vitro* and *in-silico* tools. The results of this study imply that PDL plays a crucial role in the emergence of the behavioral differences between a tooth-PDL-bone and a dental implant-bone construct, under the impact loading condition. Moreover, the results of this work can help one conclude that the bone in the dental implant-bone construct is more susceptible to mechanical damage than that of a natural tooth construct. It is hoped that results of this study can be used as a basis to facilitate future studies, and help development of dental restorative systems with improved damping capacity, which might allow maintaining tooth natural behavior and durability. In conclusion, through appreciating the vital role of the PDL in the tooth-PDL-bone construct, bioengineers might need to delve into more details of such studies to discover new aspects of it, with the ultimate goal of making a new generation of dental implants, i.e., bio-mimicked implants, which shows less deviation from a natural tooth behavior.

## Data Availability Statement

The datasets generated for this study are available on request to the corresponding author.

## Ethics Statement

The animal study was reviewed and approved by Committee on Animal Care at Faculty of Veterinary Medicine at the University of Tehran.

## Author Contributions

AK made substantial contributions to the conception or design of the work; the acquisition, analysis, and interpretation of data; development of finite element models; and drafted the work. GR made substantial contributions to the conception or design of the work; interpretation of data; revision of the work critically for important intellectual content. MD made substantial contributions to the conception or design of the work; revision of the work critically for important intellectual content. SF and HB made substantial contributions to the conception or design of the work; conducted experimental procedure; revised the work critically for important intellectual content.

## Conflict of Interest

The authors declare that the research was conducted in the absence of any commercial or financial relationships that could be construed as a potential conflict of interest.
